# Experimental Study on the Localized Deformation and Damage Behavior of Polymer-Bonded Explosive Simulant under Cyclic Compression

**DOI:** 10.3390/ma17040919

**Published:** 2024-02-16

**Authors:** Dong Jia, Zhiming Hao, Yunqiang Peng, Shunping Yan, Wenjun Hu

**Affiliations:** 1 Institute of Systems Engineering, China Academy of Engineering Physics, Mianyang 621999, China; caepjd@163.com (D.J.); peng.rong.qiang@163.com (Y.P.); yanshunping@139.com (S.Y.); wjhu@vip.sina.com (W.H.); 2 Shock and Vibration of Engineering Materials and Structures Key Laboratory of Sichuan Province, Mianyang 621999, China

**Keywords:** polymer-bonded explosive, uniaxial cyclic compression, computed tomography, digital volume correlation, shear localization

## Abstract

Uniaxial cyclic compression tests were performed to investigate the compression deformation and damage of polymer-bonded explosive (PBX) simulant, particularly shear localization. The macroscopic mechanical behavior and mesoscale failure mechanisms of the PBX simulant were analyzed by optical observation and SEM scanning methods. After each cyclic compression, the specimen was scanned by X-ray computed tomography (CT), and the internal 3D deformation of the specimen was calculated using the digital volume correlation (DVC) method. The results show that the stress–strain curve of the PBX simulant exhibits five stages and coincides with the morphological changes on the surface of the specimen. The mesoscale failure mechanism is dominated by particle interface debonding and binder tearing, accompanied by a small amount of particle breakage. There are three bifurcation points (T_1_, T_2_, and T_3_) in the curves of the normal and shear strain components with compression strain. It was found that these bifurcation points can reflect the full progression of the specimen from inconspicuous damage to uniformly distributed damage, shear localization, and eventual macroscopic fracture. The strain invariant *I*_1_ can quantitatively and completely characterize the deformation and damage processes of the PBX simulant under cyclic compression.

## 1. Introduction

Polymer-bonded explosives (PBXs) have the advantages of high safety, easy processing, and good strength, and they are widely used in weaponry and civilian fields. PBXs are multiphase composite materials composed of high-energy explosives particles of irregular shapes, different sizes, and random position distributions (e.g., HMX (octahydro-1,3,5,7-tetranitro-1,3,5,5,7-tetrazocine), TATB (1,3,5-triamino-2,4,6-trinitrobenzene)) and polymer binders, as well as plasticizers, passivators, etc. The weight fraction of explosive particles in PBXs is up to 90% or more, e.g., the weight fraction of TATB explosive grains in PBX 9502 is 95% [1]. In the microstructure, the size of the explosive particles in PBXs exhibits a broad-spectrum distribution, which ensures a high volume fraction of explosive particles, to meet the blast energy requirements of explosives in a weapon system. In the course of weapon service, PBX components may be deformed and damaged under the action of external loads, which can also lead to destruction in serious cases. This will affect the explosive blast performance of explosives, and finally affect the operational effectiveness of the weapon. Therefore, the study of the damage and fracture behavior of PBX materials is of great significance for weapon reliability [2,3,4,5].

There is some understanding in the study of PBX material damage and fracture behavior; macroscopic fracture modes are dominated by tensile and shear fracture, and microscopic damage mechanisms are dominated by interface debonding, binder tearing, particle crushing, etc. [6,7,8,9,10]. However, these studies are mainly based on surface observation and fracture surface analysis of the specimens, which cannot reveal the internal damage behavior of PBXs during loading [9]. With the development of ray detection technology, X-rays have been widely used in the study of the mechanical properties of explosives and their crystal particles [11,12,13]. Researchers also applied X-ray computed tomography (CT) technology to study the internal structure characteristics and defects of PBXs. These include microstructure changes under indirect tensile stress [14], pore changes under thermal cycling [15], microstructure fracture behavior under compression [16], interface damage and healing behavior between explosive particles and binders [17], etc. Although CT techniques have been widely used in mesoscale experimental studies of PBXs, it is difficult to quantitatively characterize the internal deformation and damage distribution of PBXs by CT techniques alone [18,19].

With the development of digital volume correlation (DVC) technology, some researchers have developed a CT-based DVC analysis method, which can quantitatively characterize the internal damage of materials by calculating the internal 3D displacement and strain fields [20,21,22,23]. Hu et al. [24] and Xu et al. [25] combined CT and DVC techniques to investigate the internal structure and deformation characteristics of a PBX simulant under compression loading but did not quantitatively analyze the complete evolution laws of the strain field and shear localization behavior. Wang et al. [26] also used this technique to study the in situ compression damage behavior of TATB-based PBXs, obtaining the 3D displacement and strain fields before the peak load, but did not investigate the strain localization behavior after the peak load.

Shear fracture under compression loading is an important failure mode of PBXs. The compression test method is of great significance for damage modeling and the fracture criteria establishment for PBXs. At present, researchers have carried out numerous studies on the damage evolution process of PBXs before the compression peak point using the CT-based DVC method. However, complete quantitative experimental studies on deformation and damage after the compression peak, especially the shear localization process, are lacking.

In this paper, a research methodology combining cyclic compression loading–unloading experiments with CT scans and DVC calculations was first presented, which was used to study the internal deformation and damage of the PBX simulant. The macroscopic mechanical behavior and mesoscale failure mechanisms of the PBX simulant were obtained. By analyzing experimental results and DVC calculations, the key bifurcation points on the evolution curves of the strain components were found, which are related to the shear localization behavior of PBX simulants under cyclic compression. A reasonable method for describing the shear localization behavior of PBX simulants was proposed. The method uses the strain invariant *I*_1_ to characterize the complete deformation and damage process of PBX simulants under cyclic compression.

## 2. Materials and Methods

### 2.1. Materials and Specimen

**Figure 1:**
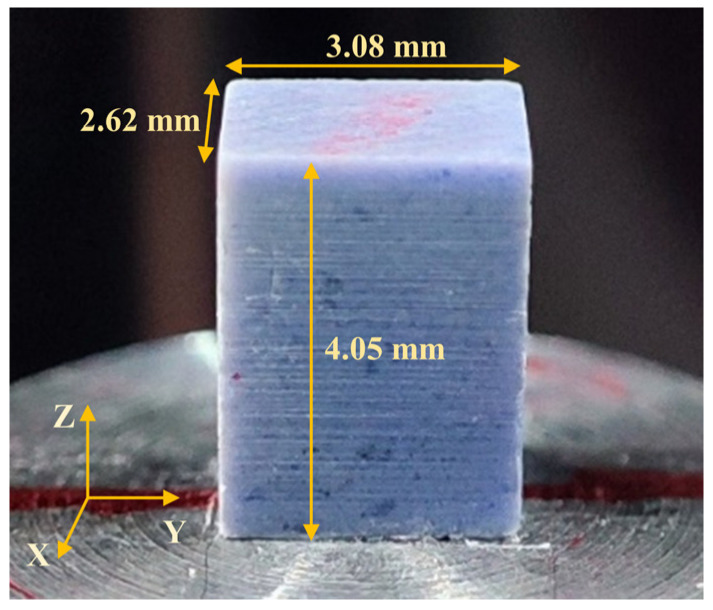
The specimen of PBX simulant.

The material used in this study was a PBX simulant, in which a combination of two inert crystals, barium nitrate and melamine, was used in place of the TATB/HMX crystals in the PBX. The weight percentages of the crystal particles and the binder were above 90% and below 10%, respectively, meaning that the density of the PBX simulant was close to that of the PBX. Moreover, the same binder and molding processes were employed. This basically ensured the consistency of the mechanical properties of the PBX simulant with those of the PBX and met the requirements for substitution in terms of mechanical properties. The dimensions of the specimen were 4.05 mm × 3.08 mm × 2.62 mm, and the front–back direction of the specimen was direction X, the left–right direction was direction Y, and the top–bottom direction was direction Z, as shown in Figure 1.

### 2.2. Cyclic Compression and CT Scanning Experiments

**Figure 2:**
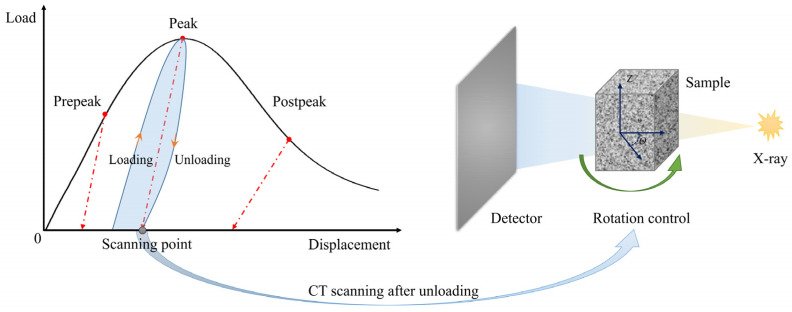
Schematic diagram of cyclic loading and unloading with CT scanning.

The PBX simulant specimen was subjected to the cyclic compression loading and unloading tests with a displacement-controlled mode. The displacement loading and unloading rate was 1 μm/s. The loading direction was in direction Z. As shown in Figure 2, before reaching the peak load, an incremental increase of 20 N was performed for each loading, and unloading was carried out when the load reached the maximum value in each cycle. After reaching the peak load, since the specimen showed compression–softening behavior, the maximum load per cycle could not be accurately controlled by force. When each cyclic loading–unloading experiment was completed, the specimen was placed on the specimen stage of the CT unit for scanning. The X-ray for CT scanning adopted a Cu target with a scanning resolution of 6 μm and a rotation angle of 0.4°, as shown in Figure 2. Under compression, the particles and binder in the specimen will deform, and defects like initial cracks and voids within the specimen will gradually develop. These changes lead to the overall deformation of the specimen. From a macroscopic point of view, the deformation of the specimen consists of the reversible and irreversible deformations. After unloading, the reversible deformation of the specimen will disappear due to the recovery of the elastic deformation and the closure of some cracks and voids, while the irreversible deformation will remain. In this paper, the internal morphology of the specimen after each unloading is obtained by CT scanning, and the irreversible deformation is calculated by DVC in order to study the damage of the PBX simulant after each cycle of compression.

## 3. The Augmented Lagrangian Digital Volume Correlation Methodology

The digital volume correlation (DVC) methods were proposed in the late 1990s [27]. Assume a three-dimensional spatial domain denoted as Ω. And the voxel’s reference coordinate is represented by X. The position is described by y(X). The relationship between the initial scattering field and the corresponding scattering field with grey values of f(X) and g(y), respectively, can be expressed as

(1) f(X)=g(y(X))

By establishing a unique mapping relationship between the reference image and the deformation image, the corresponding displacement and strain fields can be calculated.

DVC methods include local DVC methods (Local-DVC) and global DVC methods (Global-DVC). The main difference between the two kinds of methods is that the boundary displacements of neighboring subsets are discontinuous in Local-DVC, while in Global-DVC, the node displacements of neighboring cells are continuous [28]. So the computational efficiency of Local-DVC is higher, and the computational accuracy of Global-DVC is higher [29]. In 2020, Yang et al. [30] proposed the Augmented Lagrangian Digital Volume Correlation (ALDVC) method, which combines the advantages of the high computational efficiency of Local-DVC and the high accuracy of Global-DVC. Considering that the full-field displacements should satisfy the kinematic compatibility constraints, the ALDVC method introduces an auxiliary compatible global displacement field $\hat{u}$ that satisfies

(2) $$\begin{array}{l} F_{i}=\nabla \hat{u}(X_{i0})\\ u_{i}=\hat{u}(X_{i0}) \end{array}$$

where $X_{i0}$ is the centroid of a local subset $\Omega_{i}$, $u_{i}$ is the displacement of $X_{i0}$, and $F_{i}$ is the uniform displacement gradient of $\Omega_{i}$. The global constraint is defined by augmenting the Lagrangian form, which is included in the correlation function as

(3) $$\begin{array}{ll} {ℒ}_{0} & =\sum_{i}\int_{\Omega_{i}}({|f(X)-g(X+u_{i}+(F_{i}(X-X_{i0})))|}^{2}\\ & +\frac{\beta }{2}|{\left(D\hat{u}\right)}_{i}-F_{i}+W_{i}{|}^{2}+\frac{\mu }{2}|{\hat{u}}_{i}-u_{i}+v_{i}{|}^{2})dX \end{array}$$

where $W_{i}$ and $v_{i}$ are Lagrange multipliers used to augment the constraints in Equation (2). β and µ are two coefficients. Based on the Alternating Direction Multiplication Method (ADMM), the ALDVC method improves the efficiency of the global iterative solution by dividing the global maximization problem into independent subdomains [30].

Based on the three-dimensional displacement field given by the ALDVC method, the Green–Lagrangian strain tensor E can be calculated by the finite difference method, which is defined as

(4) $$E=\frac{1}{2}(C-I)=\frac{1}{2}(F^{T}F-I)$$

where C is the right Cauchy–Green strain tensor, and I is the unit strain tensor. Additionally, the strain components $\varepsilon_{ij}(i,j=1,2,3)$ of the strain tensor E can be described as

(5) $$\varepsilon_{ij}=\frac{1}{2}\left(C_{ij}-\delta_{ij}\right)=\frac{1}{2}\left(\frac{\partial u_{i}}{\partial X_{j}}+\frac{\partial u_{j}}{\partial X_{i}}+\frac{\partial u_{k}}{\partial X_{i}}\frac{\partial u_{k}}{\partial X_{j}}\right)$$

According to the symmetry ($\varepsilon_{12}=\varepsilon_{21}$, $\varepsilon_{13}=\varepsilon_{31}$, $\varepsilon_{23}=\varepsilon_{32}$) of the strain components, there are only six independent strain components. These include three normal strain components ($\varepsilon_{11}$, $\varepsilon_{22}$, $\varepsilon_{33}$) and three shear strain components ($\varepsilon_{12}$, $\varepsilon_{13}$, $\varepsilon_{23}$). For the strain tensor E, the strain invariants can be defined by

(6) $$\begin{array}{l} I_{1}=\mathrm{trace}\left(E\right)\\ I_{2}=\frac{1}{2}\left(\mathrm{trace}{\left(E\right)}^{2}-\mathrm{trace}\left(E^{2}\right)\right)\\ I_{3}=\det\left(E\right) \end{array}$$

where $I_{1}$, $I_{2}$, and $I_{3}$ represent the first, second, and third invariants of the strain tensor, respectively. The trace() means the trace of the tensor, and det() means the determinant of the tensor. The first invariant $I_{1}$ represents the extent of material expansion or contraction, whereas the second invariant $I_{2}$ and the third invariant $I_{3}$ are associated with the shear deformation and material distortion.

## 4. Results and Discussion

### 4.1. Macroscopic Mechanical Behaviors and Mesoscale Failure Mechanisms

**Figure 3:**
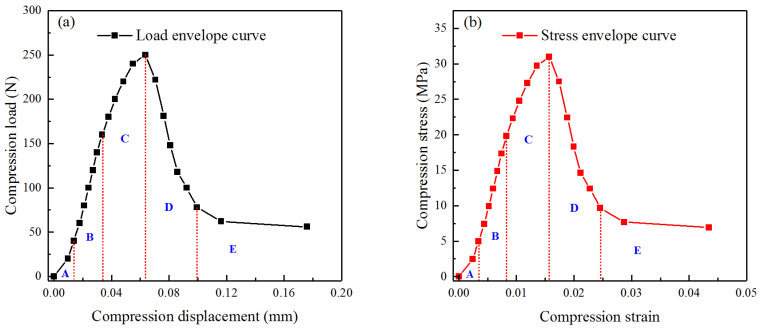
Envelope curves of cyclic compression: (**a**) load vs. displacement and (**b**) stress vs. strain. The envelope curves are divided into five stages as stage A, B, C, D, and E.

Conducting the cyclic compression loading–unloading tests, the loading and displacement values at each cyclic compression maximum load point were obtained. And the nominal stress and nominal strain of the PBX simulant at each maximum load point were calculated. All the points were connected to form the envelope of the uniaxial compression nominal stress–strain relationship of the PBX simulant, as shown in Figure 3.

**Figure 4:**
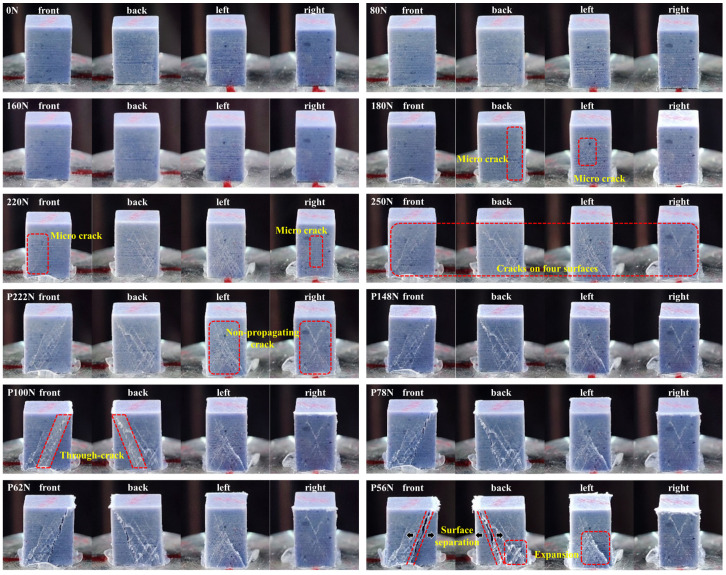
Macroscopic morphology of the specimens after cyclic compression loading and unloading (with P as post-peak load).

As shown in Figure 3b, the uniaxial compression nominal stress–strain curve for the PBX simulant can be divided into five stages. In stage A, the stress increases gradually with a nonlinear behavior. Subsequently, in stage B, the stress increases almost linearly with strain. Then, in stage C, the stress increases nonlinearly with a slowdown rate. After reaching the peak stress, stage D displays a rapid decline in stress levels. Finally, the stress level enters a relatively stable state in stage E. To show the differences among the five states, the macroscopic surface morphologies of the specimen after cyclic compression at typical loading levels are given in Figure 4.

It can be seen from Figure 4, in stages A and B (0N~160N), the surface morphology of the specimen is basically unchanged, and no cracks appear. In stage C (180N~250N), cracks first appeared on the back and left side of the specimen. Subsequently, cracks emerged on the front and right side of the specimen. These cracks gradually expanded with the increase in load. Cracks had grown significantly on all four surfaces after unloading for the peak load (250N). In stage D (P222N~P100N), the cracks on the front and back surfaces of the specimen expanded rapidly, developed into the main crack, and ran through the entire specimen after unloading at P100N. In this process, the cracks on the left and right surfaces of the specimen showed almost no growth, which indicates that the energy of the cyclic loading was mainly dissipated by the expansion of the main crack. In stage E (P78N~P56N), the penetrating cracks on the front and back surfaces of the specimen were formed, and these cracks continued to separate during the cyclic loading, forming macroscopic fracture surfaces. The formation of the macroscopic fracture surfaces was accompanied by rotational instability, which resulted in stress concentration at the end of the specimen. In the end, the local surface of the specimen expanded outward, leading to an expansion fracture in the end region of the specimen.

As shown in Figure 4, the macroscopic fracture surfaces occurred in the Y-Z plane in this paper. However, the fracture behavior of the specimen is influenced by various factors, such as the geometry of the specimen, the randomly distributed defects, and particle binder interfacial properties. When subjected to compressive loading, these mechanisms interact in a complex manner. Theoretically, the damage (cracks, voids, etc.) within the specimen may eventually concentrate in different planes (X-Z plane or Y-Z plane) to form the macroscopic shear fracture surfaces.

**Figure 5:**
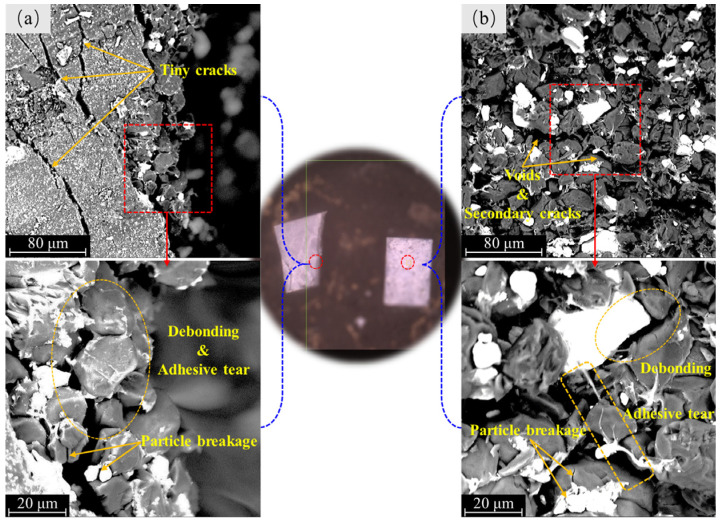
Fracture morphology of the specimen by SEM: (**a**) the junction region on the left half of the specimen, and (**b**) the macro fracture surface on the right half of the specimen.

Figure 5 illustrates the SEM observations of the macroscopic fracture surfaces. Figure 5a shows the morphology of the junction region between the macroscopic fracture surface and the left half of the specimen. Many tiny cracks can be observed close to the macroscopic fracture surface, with some oriented in the same direction as the macroscopic fracture surface and others aligned at an angle to the macroscopic fracture surface. From the examination of the magnified local area, it can be seen that the particle interface debonding and binder tearing at the edge of the macroscopic fracture surface are obvious, accompanied by a small amount of particle breakage. Figure 5b shows the morphology of the right half of the specimen on the macroscopic fracture surface. The densely distributed particles with voids and secondary cracks are presented. Further magnification reveals that these voids and secondary cracks are mainly caused by debonding at the particle interface, confirmed by the binder pulling observed in the local region. A small amount of particle breakage is also observed. Overall, the mesoscale failure mechanism of the PBX simulant under compression loading is primarily dominated by along-crystal fracture, while through-crystal fracture characteristics are relatively insignificant. This suggests that the tension effect occurring at the mesoscale of the specimens under compression loading plays an important role in the fracture of the specimens.

### 4.2. Three-Dimensional Deformation and Damage

**Figure 6:**
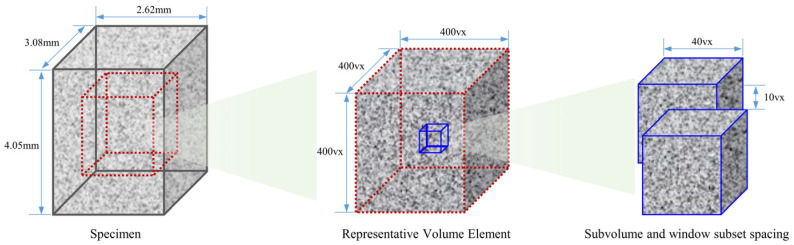
Schematic of the digital volume correlation (DVC) calculation region.

The PBX simulant mainly consists of two random distributions of particles with different densities, resulting in a grey-scale image that exhibits a markedly inhomogeneous distribution. This inhomogeneous distribution provides a natural scattering field for the digital volume correlation (DVC) calculations. In this case, a 3D representative volume element of 400 vx × 400 vx × 400 vx (vx: voxel) is selected from the central region of the specimen. The size of the digital volume correlation subunit is set to 40 vx × 40 vx × 40 vx, and the window move step is set to 10 vx × 10 vx × 10 vx, as shown in Figure 6.

**Figure 7:**
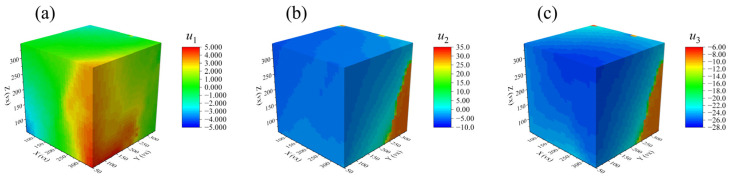
Distributions of displacement components of the specimen: (**a**) $u_{1}$, (**b**) $u_{2}$, and (**c**) $u_{3}$.

**Figure 8:**
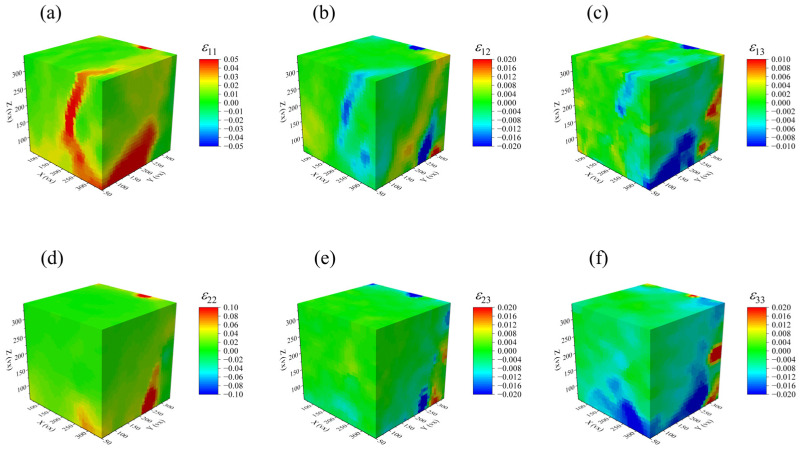
Distributions of strain components of the specimen: (**a**) $\varepsilon_{11}$, (**b**) $\varepsilon_{22}$, (**c**) $\varepsilon_{33}$, (**d**) $\varepsilon_{12}$, (**e**) $\varepsilon_{13}$, and (**f**) $\varepsilon_{23}$.

Based on the ALDVC calculation results, the normal strain components and shear strain components of the RVE were obtained. Figure 7 and Figure 8 give the three-dimensional displacement and strain fields (directions X, Y, and Z correspond to 1, 2, and 3, respectively) at P56N for a region size of 280 vx × 280 vx × 280 vx in the RVE, which fulfills the requirements of digital volume correlation calculations.

**Figure 9:**
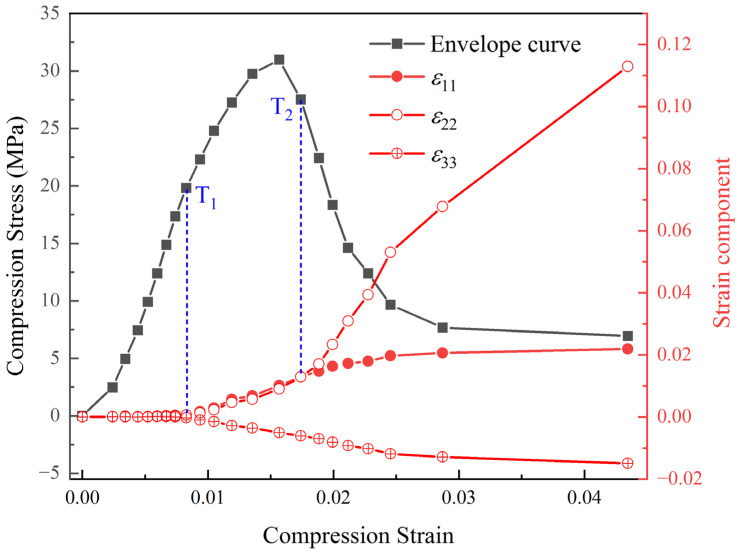
Evolution curves of normal strain components under cyclic compression.

**Figure 10:**
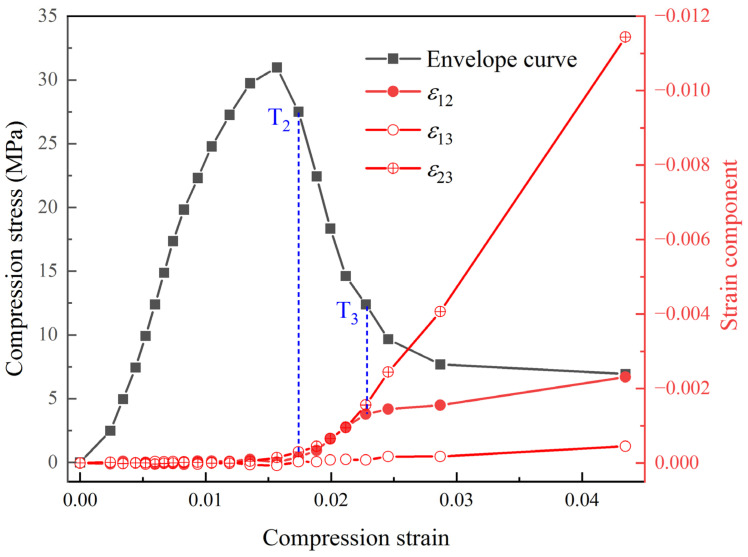
Evolution curves of shear strain components under cyclic compression.

In Figure 7 and Figure 8, the shear localization behavior appeared in the specimen subjected to uniaxial compression loading. This behavior is reflected in the final 3D displacement field and strain field, being consistent with the macroscopic damage mode of the specimen. By calculating the average values of the strain components at different locations in the 3D space of the RVE, the variation patterns of the normal strain and shear strain components in the 3D strain field for the PBX simulant with cyclic compression loading are shown in Figure 9 and Figure 10. The results indicate that the shear strain components are all less than or equal to 0. And the absolute values of the shear strain are given in Figure 10 for convenience of comparative analysis.

Figure 9 shows the relationship between the normal strain component and compression strain. It reveals that there are two obvious bifurcation points, namely T_1_ (compression strain of 0.008, corresponding to the load of 160N), and T_2_ (compression strain of 0.017, corresponding to the load of P222N) on the evolution curve of normal strain component. Before the T_1_ point, the three normal strains ($\varepsilon_{11}$, $\varepsilon_{22}$, and $\varepsilon_{33}$) are close to zero, indicating negligible irreversible deformation inside the specimen. Between T_1_ and T_2_, the three normal strains ($\varepsilon_{11}$, $\varepsilon_{22}$, and $\varepsilon_{33}$) show different trends. The normal strains $\varepsilon_{11}$ and $\varepsilon_{22}$ are greater than zero and increase gradually. $\varepsilon_{33}$ is less than zero and increases gradually. It indicates that irreversible deformation occurs uniformly inside the specimen, suggesting that the evolution of defects such as cracks and holes presents a random and dispersed state within the specimen. Beyond T_2_, the evolution curves of normal strain components are also different. The changes in normal strain components $\varepsilon_{11}$ and $\varepsilon_{33}$ slow down, while the normal strain component $\varepsilon_{22}$ increases rapidly. This indicates that the deformation after T_2_ is mainly concentrated on the normal strain component $\varepsilon_{22}$, which transforms from the homogeneous and dispersed state to the localized state. And the cracks and holes developed to converge into the main cracks. This is corroborated with the crack extension phenomenon observed on the specimen surface, as sketched in Figure 4.

Figure 10 shows the relationship between the shear strain component variation and compression strain. It displays that there are two bifurcation points, namely T_2_ (consistent with T_2_ in the normal strain component of Figure 9) and T_3_ (compression strain of 0.023, corresponding to the load of P100N) in the evolution curve of shear strain components. Before T_2_, the three shear strain components ($\varepsilon_{12}$, $\varepsilon_{13}$, and $\varepsilon_{23}$) are close to 0, indicating minimal shear deformation occurring inside the specimen. Instead, the irreversible deformation of the specimen is dominated by volume deformation. Between T_1_ and T_2_, the shear strain components are still close to 0, even though the irreversible normal strain components developed in the specimen. At this stage, the homogeneous deformation is still uniform, and the gradient of the displacement values in non-identical directions is zero, i.e., $\partial u_{i}/\partial X_{j}=0,\begin{array}{c} i\neq j \end{array}$. According to the definition of the Green–Lagrangian strain tensor (Equations (4) and (5)), it can be derived that all shear strain components $\varepsilon_{ij}(\begin{array}{c} i\neq j \end{array})$ are zero. Based on the evolution curves of the normal and shear strain components, it is concluded that the specimen shows diffuse damage between the bifurcation transition points T_1_ and T_2_, while the localized damage is not apparent.

Between T_2_ and T_3_, the shear strain components show different trends. The absolute values of shear strains $\left|\varepsilon_{12}\right|$ and $\left|\varepsilon_{23}\right|$ increase gradually, while $\left|\varepsilon_{13}\right|$ is still close to zero. This indicates that the specimen not only undergoes volume deformation, but also shape deformation in the X-Y and Y-Z planes. As a result, the shear localization behavior becomes increasingly noticeable. Beyond T_3_, the shear strains $\left|\varepsilon_{12}\right|$ and $\left|\varepsilon_{23}\right|$ are different. The change in $\left|\varepsilon_{12}\right|$ tends to slow down, while $\left|\varepsilon_{23}\right|$ still increases rapidly. It indicates that the shear deformation of the specimen is concentrated on the Y-Z plane, forming the macroscopic fracture surface. In addition, the increase in $\left|\varepsilon_{23}\right|$ can be attributed to two factors, including the gradual separation of the macroscopic fracture surface and the rotational instability of the block formed after the fracture of the specimen. 

**Figure 11:**
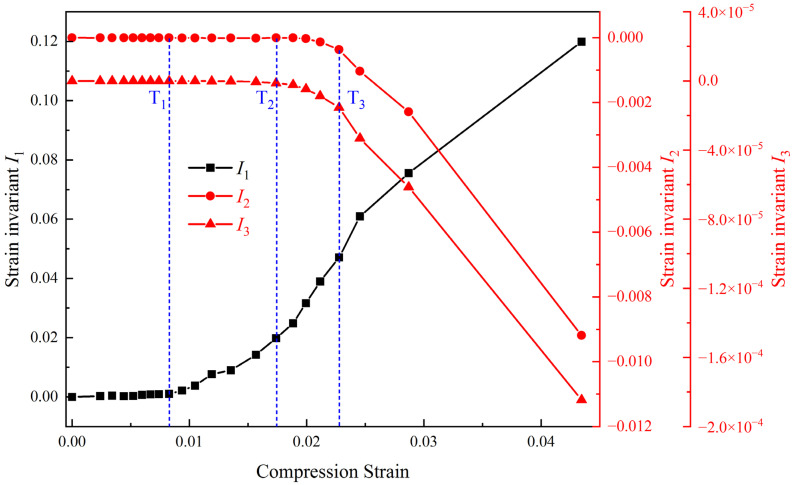
Evolution curves of strain invariants with cyclic compression strain.

Based on the results of the strain components, the strain invariants of the representative volume element were calculated by Equation (6). Figure 11 shows the variation in the three invariants ($I_{1}$, $I_{2}$, and $I_{3}$) with cyclic compression loading. Also, Figure 11 shows the correlation relation between the bifurcation points T_1_, T_2_, and T_3_ of the strain component curves and the changes in strain invariants $I_{1}$, $I_{2}$, and $I_{3}$. Before T_1_, all strain invariants remain close to 0, indicating no significant volume change or shape change in the specimen. Between T_1_ and T_2_, the strain invariant $I_{1}$ gradually increases, while the strain invariants $I_{2}$ and $I_{3}$ show little change, suggesting that the deformation of the specimen is dominated by volumetric expansion at this stage. Between T_2_ and T_3_, the first strain invariant $I_{1}$ continues to increase rapidly, while the second strain invariant $I_{2}$ and the third strain invariant $I_{3}$ decrease slowly. This indicates that the volume expansion deformation is still dominated between T_2_ and T_3_, but the deformation uniformity cannot be maintained. After T_3_, the first strain invariant $I_{1}$ still continues to increase, while the other two strain invariants $I_{2}$ and $I_{3}$ both decrease rapidly, indicating that the volume expansion of the specimen is accompanied by a pronounced shape change. The result coincides with the macroscopic fracture surface separation and rotational destabilization phenomenon in the final stage. So, the trends of the strain invariants are consistent with that of the strain components.

**Figure 12:**
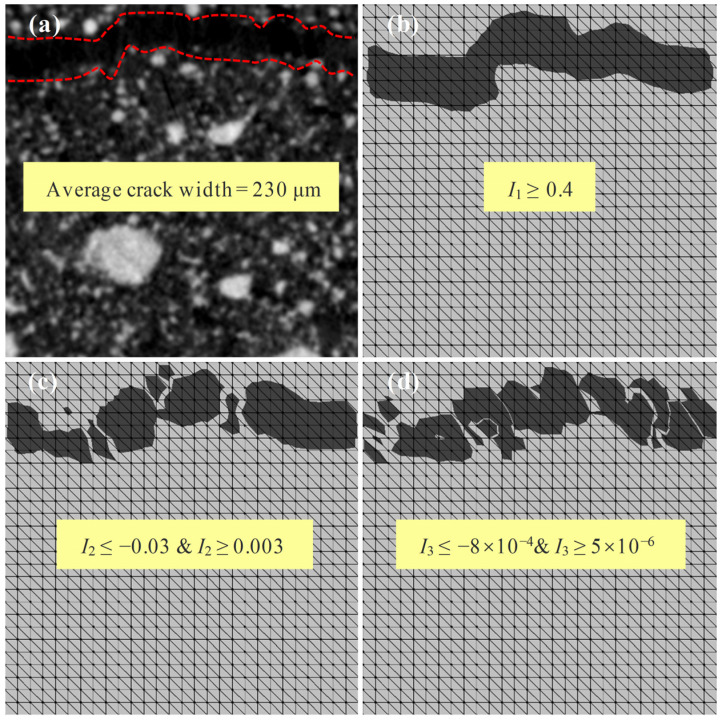
Comparison of crack patterns obtained from experiments (**a**) with those obtained from calculations at the thresholds of strain invariants (**b**) *I*_1_, (**c**) *I*_2_, and (**d**) *I*_3_.

Figure 12 shows the characterization results of the crack morphology for different strain invariants. The CT image at the P56N load level revealed an average width of 230 μm for the macroscopic main crack. By comparing the cracks observed in the CT images with the macroscopic cracks calculated by ALDVC (the area between the red dotted lines in Figure 12), the threshold values of strain invariants for characterizing the macroscopic main cracks were determined, where $I_{1}\geq 0.4$, $I_{2}\leq -0.03\&I_{2}\geq 0.003$, and $I_{3}\leq -8\times 10^{-4}\&I_{3}\geq 5\times 10^{-6}$.

As can be seen from Figure 12, the morphology of the macroscopic main crack in the specimen is better characterized by the first strain invariant $I_{1}$, whereas the macroscopic main crack characterized by the second and third strain invariants $I_{2}$ or $I_{3}$ is incoherent and differs more significantly from the actual crack morphology observed in the experiment. In order to further investigate the deformation and damage process of the PBX simulant under cyclic compression, the first strain invariant $I_{1}$ was used as a characteristic variable to conduct the deformation and damage distributions of the specimens.

**Figure 13:**
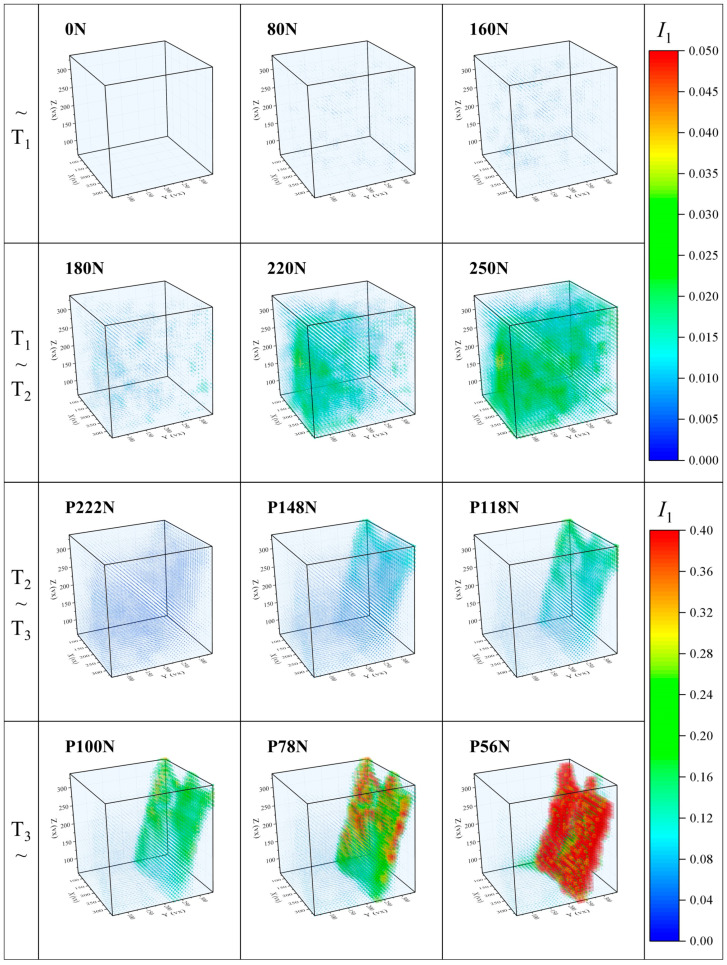
Cloud maps of the three-dimensional distribution of strain invariant *I*_1_.

Since the first strain invariant $I_{1}$ varies greatly during the compression experiments, the whole evolution of the first strain invariant $I_{1}$ is analyzed by relating the characteristic bifurcation points T_1_, T_2_, and T_3_ stage by stage. As shown in Figure 13, the rainbow cloud maps representing the values of first strain invariant $I_{1}$ from 0 to 0.05 are used in the two stages of ~T_1_ and T_1_~T_2_, while the rainbow cloud maps representing the values of the first strain invariant $I_{1}$ from 0 to 0.4 are used in the two stages of T_2_~T_3_ and T_3_~.

Figure 13 reveals that the cloud map of the first strain invariant $I_{1}$ distributed in 3D space contains valuable information about the internal deformation characteristics. Before T_1_, the strain invariant $I_{1}$ is relatively small and uniformly distributed in the specimen. Between T_1_ and T_2_, the first strain invariant $I_{1}$ starts to increase rapidly and remains relatively uniformly distributed. Between T_2_ and T_3_, the first strain invariant $I_{1}$ begins to show localized concentration, gradually intensifying over time. This phenomenon becomes evident when the main crack surface exhibits a shear morphology. After T_3_, the deformation and damage, represented by $I_{1}$, are mainly concentrated on the main crack surface, and further develop to form a macroscopic fracture surface. The deformation and damage of the specimen are directly related to the evolution of the first strain invariant $I_{1}$. Therefore, based on the first strain invariant $I_{1}$, the formation process of localized damage and macroscopic fracture modes of PBX simulant under compression loading can be effectively revealed.

In this paper, the CT-based DVC method was introduced to investigate the cyclic loading–unloading behavior of PBX simulant. It successfully captures the irreversible deformation of the specimen after each compression cycle, providing a visual representation of the damage degree. Moreover, CT scanning of the specimen after unloading helps avoid persistent damage or fracture that may occur when the specimen remains compressed for a long time, especially when it enters a destabilized state after the peak load. However, the methodology used in this paper also has certain limitations. In order to assess the overall deformation and damage evolution during compression, the specimen must be of sufficient size to be representative. To ensure the integrity of the scanning field of view range, the resolution of the CT scan cannot be too high, which results in defects within the material being visually recognized. In addition, the closure or recovery of the cracks and voids after unloading adds to the challenge of identifying microscopic defects by CT scanning. To address these limitations, it is necessary to carry out in situ analyses of the microscopic defects for PBX simulants in the future by using higher-resolution ray scanning devices (e.g., synchrotron radiation light sources, etc.) or numerical simulations.

## 5. Conclusions

In conclusion, this paper investigates the mechanical behavior of PBX simulant under uniaxial cyclic compression. The analysis covers the macroscopic deformation, mesoscale fracture mechanism, internal 3D deformation, and damage behavior. The main findings are summarized as follows:

(1) Under uniaxial cyclic compression, the compression stress and macroscopic morphology of the PBX simulant can be divided into five stages. The mesoscale failure behavior is dominated by particle interface debonding and binder tearing, accompanied by a small amount of particle breakage.

(2) The evolution curves of the normal and shear strain components exhibit three obvious bifurcation points (T_1_, T_2_, and T_3_). The bifurcation points T_1_ and T_2_ indicate the evolution of the deformation and damage of the specimen from a uniform state to a localized state. The bifurcation points T_2_ and T_3_ depict the evolution process from localized deformation and damage to macroscopic fracture.

(3) The bifurcation points (T_1_, T_2_, and T_3_) exhibit a close correlation with the trend of the strain invariants. And the complete deformation and damage process of the PBX simulant under cyclic compression can be effectively characterized by the first strain invariant $I_{1}$.

## Data Availability

Data are contained within the article.
